# Male involvement enhances the uptake of early infant diagnosis of HIV services in Thyolo, Malawi: A non-equivalent control group quasi-experimental study

**DOI:** 10.1371/journal.pone.0281105

**Published:** 2023-02-22

**Authors:** Miriam Window, Alinane Linda Nyondo-Mipando, Noel Kalanga

**Affiliations:** 1 Department of Health Systems and Policy, School of Public Health and Family Medicine, Kamuzu University of Health sciences (Formerly College of Medicine), Blantyre, Malawi; 2 Department of Reproductive Health, School of Maternal, Neonatal and Reproductive Health, Kamuzu University of Health Sciences (Formerly College of Nursing), Blantyre, Malawi; Elizabeth Glaser Pediatric AIDS Foundation, UNITED STATES

## Abstract

**Background:**

Poor retention of HIV-exposed infants (HEIs) in the Early Infant Diagnosis (EID) programme remains a significant challenge and impedes progress towards the elimination of Mother to Child Transmission (eMTCT). Suboptimal involvement of a father in his child’s participation in the EID of HIV services is one of the reasons for delayed initiation and poor retention in EID. This study compared the uptake of EID of HIV services at 6weeks from 6 months pre and post-implementation of the Partner invitation card and Attending to couples first (PA) strategy for male involvement (MI) at Bvumbwe Health Centre in Thyolo, Malawi.

**Methods:**

We conducted a non-equivalent control group quasi-experimental study from September 2018 to August 2019 and enrolled 204 HIV positive women with HIV exposed infants who delivered at Bvumbwe health facility. 110 women were in the period before MI in EID of HIV services from September 2018 to February 2019 whereas 94 of them were in the period of MI in EID of HIV services from March to August 2019 receiving PA strategy for MI. Using descriptive and inferential analysis we compared the two groups of women. As age, parity and education levels of women were not associated with the uptake of EID, we proceeded to calculate unadjusted odds ratio.

**Results:**

We observed an increase in the proportion of women that took up EID of HIV services such that 64/94 (68.1%) came for EID of HIV services at 6weeks from 44/110 (40%) in the period before MI. The uptake of EID of HIV services had an odds ratio of 3.2(95%CI: 1.8–5.7) P = 0.001) compared to the uptake of EID of HIV services before MI OR of 0.6(95%CI: 0.46–0.98) P = 0.037). Age, parity, and education levels of women were statistically insignificant.

**Conclusion:**

The uptake of EID of HIV services at 6 weeks increased during the implementation of MI compared to the period before. Age, parity, and education levels of women were not associated with the EID uptake of HIV services at 6 weeks. Further studies on male involvement and uptake of EID should continue to be carried out to contribute to understanding of how high levels of EID uptake of HIV services can be achieved.

## Background

Early Infant Diagnosis (EID) of HIV is one of the crucial steps in the prevention of Mother to Child Transmission programs (PMTCT). It offers a platform for assessment, diagnosis, and linkage to treatment as necessary among HIV-exposed infants. Without early treatment of HIV/AIDS, one-third of the babies living with HIV die within one year [1]. EID of HIV has a series of activities including; collecting of blood samples, testing of HIV-exposed infant’s using dry blood spot (DBS) polymerase chain reaction (PCR) technology at 4–6 weeks of life, and transmission of test results from a laboratory to a tested infants point of care facility [2]. The goal of the EID of HIV is to optimize the detection of intrauterine, intrapartum, and early postnatal mother-to-child HIV transmission [2]. Despite the potential of EID in curbing pediatric HIV, the uptake has remained low globally at 60.3% and 72.6% in Malawi as of 2019 [3].

The major challenge with the uptake of EID of HIV is poor retention of HIV positive mothers and HIV-exposed infants (HEIs) in the PMTCT program [4–6]. The loss to follow-up ranges from 20% to 45% in Sub-Saharan Africa [7]. Male involvement is a key factor in the uptake of EID of HIV and retention in care [8]. The absence of a husband’s support [7,9] coupled with his ignorance of the baby’s HIV test results exacerbates poor uptake and retention in care. Other reasons for loss to follow-up include time and cost required for multiple visits which make women miss appointment dates [7,10–12]; young maternal age [13]; and instances when an infants’ mother is not on antiretroviral therapy (ART) before pregnancy which results into a mother being unaware of the need for early testing for her baby [10]; a poor attitude of health workers and lack of privacy at ART clinics [6,13,14]. Nonetheless, men remain critical partners in the uptake of the services.

Although MI in PMTCT yields optimal uptake of services and with evidence suggesting that uptake of EID of HIV is a challenge when a male partner is not involved [8], interventions that include male support in EID of HIV uptake are few. Men are the decision-makers in many African countries where PMTCT is offered [15] and they decide the health approaches and interventions a family will take including HIV-related decisions [16,17]. The benefits of MI in antenatal care (ANC) and PMTCT are well researched [15].

The available strategies that have been used to increase the uptake of EID of HIV services and increase MI in ANC and PMTCT have limited focus on the effectiveness of male involvement in the uptake of EID of HIV services. Some of the general strategies include Mother-to-mother mentorship programs [18,19], enhanced EID referral where women are directly accompanied by a maternity nurse before discharge to the location of EID of HIV services within the hospital grounds [20], community health worker follow up of pregnant women and their HIV exposed infant [21]; appointment reminder through mobile services [22] and integration of immunization clinic and HIV testing [23]. Given the relevance of male involvement in the uptake and retention in EID services and cognizant of the paucity of data in the area, this study compared the uptake of EID of HIV services at six weeks from 6 months pre and post-implementation of the Partner invitation card and Attending to couples first (PA) strategy for MI (This intervention has been described in detail in the conceptual framework) at Bvumbwe Health Centre in Thyolo, Malawi.

## Methods

### Study design and setting

We conducted a non-equivalent control group quasi-experimental study at Bvumbwe Health Centre in Thyolo district which is located in the southern region of Malawi between September 2018 and August 2019. We preferred this design because of its flexibility to compare uptake of EID of HIV pre and post-implementation PA strategy.

We created two cohorts that had equal follow up duration (pre-MI in EID of HIV and MI in EID of HIV period). In the pre-MI in EID HIV positive women who delivered an exposed infant from September 2018 to February 2019 were enrolled (retrospective cohort). Similarly, HIV positive women with an exposed infant at birth were also recruited from March to August 2019 (prospective cohort) and followed up to 6 weeks for EID.Infants who came after 6weeks of age post-delivery (at 7 weeks thereafter) were considered as late for EID hence not included in the study. Therefore, this study tested whether PA for MI would increase uptake of EID at 6 weeks.

### Conceptual framework

**Fig 1:**
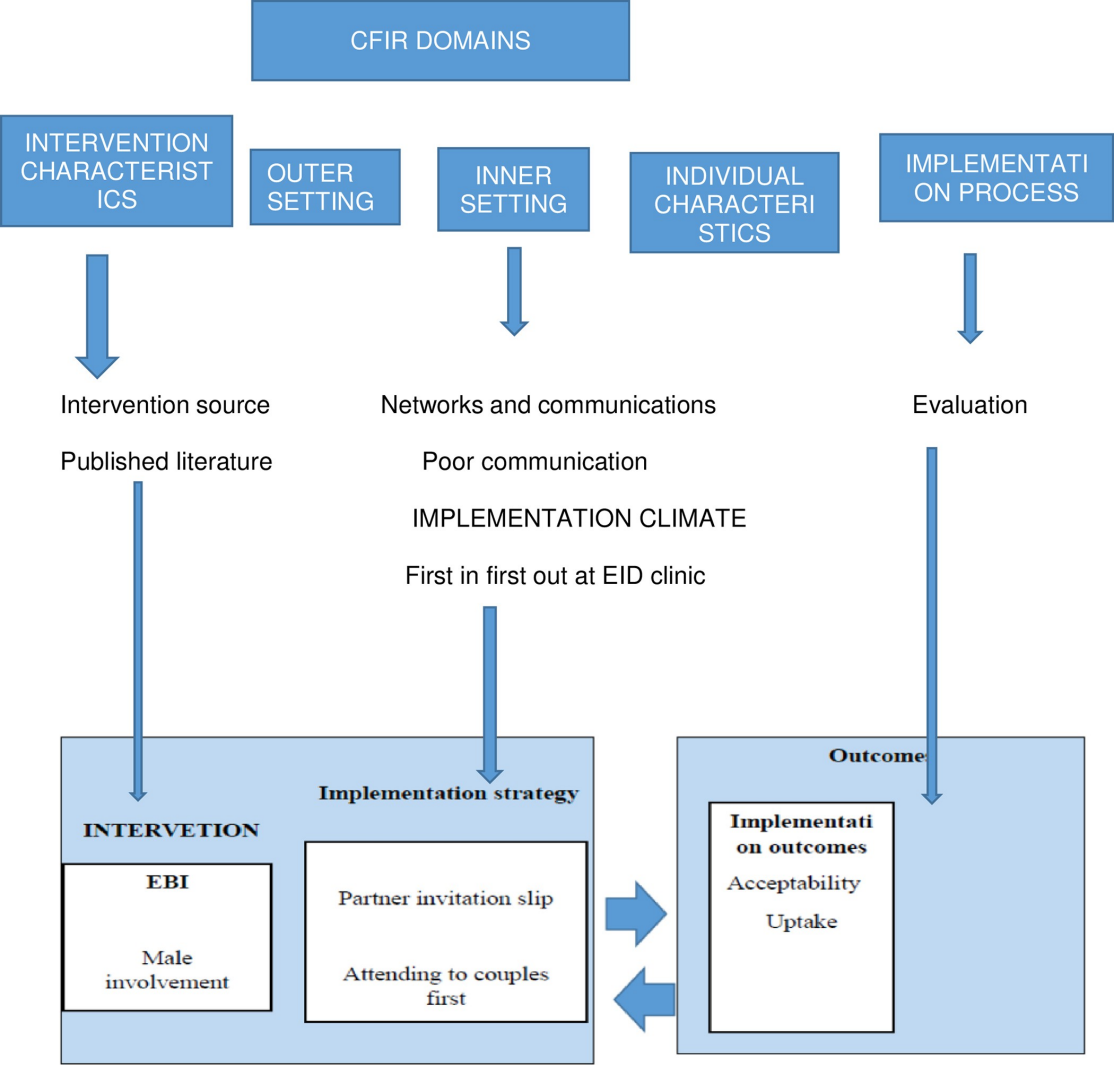
Conceptual framework that has guided the researcher to identify strategies for male involvement in EID of HIV uptake.

This study was guided by the Consolidated Framework for Implementation Research (CFIR). The framework guided us in identifying the barriers and strategies to implementation of Male involvement as an evidence-based intervention(EBI) using its constructs and domains [24]. The researcher identified MI as an EBI to help in optimizing uptake of EID of HIV. MI is an EBI which was developed by researchers to increases the uptake of maternal and child health services [16,25–28], this intervention is supported by published literature that has used robust research designs in its implementation [25,29]. We used the implementation climate and communications as constructs in the domain of the inner setting to identify barriers in EID. Therefore, we identified poor communication between the facility and the partner and the health system which considers first come first served in attending to clients or patients among others as barriers to implementing MI through literature review [30,31]. The barriers were under the inner setting as domains in the CFIR. Therefore, partner invitation was identified as a strategy to address poor communication between the healthcare worker and a male partner which is under the inner setting of the intervention. Attending to couples first was also addressing the inner setting and process of implementation by restructuring the health system to help in releasing men fast for other social-economic activities. Midwives and HIV Diagnostic Assistant (HDA) assisted us in the implementation of the strategy. The midwives were involved in communicating with men whose partners visited the facility for delivery through the use of partner invitation cards whereas the HDAs were involved in conducting EID tests on infants at six weeks. The HDAs were assisting couples first at the clinic as a way of rewarding men who accompanied their partners. Lastly, the researcher assessed the uptake of EID of HIV as an implementation outcome based on strategies used to evaluate the progress of implementation. The framework is illustrating in Fig 1.

HIV-positive women with an exposed infant in the MI in the EID of HIV period were given an invitation card as part of the PA strategy to come with their partner for EID of HIV at six weeks soon after delivery in the labour ward upon consenting.

The message on the partner invitation card was adopted from an implementation study done in Malawi on the feasibility of invitation slip in enhancing male involvement in PMTCT services [29]. The card was given to women that were eligible with instructions to extend it as a communication to their husband/partner to accompany them for EID of HIV services (S1 Appendix). At six weeks those that came as couples for EID were attended to first by an HDA to reduce waiting time for men and they were counselled on the importance of EID to reduce poor retention to care. Therefore, our primary objective compared uptake of EID of HIV at 6 weeks from 6 months pre and post-implementation of PA for MI.

### Ethical consideration

The study and all relevant documents that we used were approved by College of Medicine Research Ethics committee (COMREC) study number p.09/18/2478. Permission to conduct the study was also obtained from Bvumbwe Health Centre through the Thyolo District Health office. All eligible participants recruited in this study provided either a written/a witnessed thumb-print consent prior to study participation. The researcher only collected data that was necessary for the study. All women who refused to participate in the study were assured that their normal medical care would not be affected in any way.

### Inclusion and exclusion criteria

The study population in the pre-intervention period included records of HIV positive women who delivered at the facility with a live neonate and were registered in PMTCT/baby’s pink card was filled (Card that documents exposed infants visits to the facility). Whereas, the study population in the intervention (MI in EID of HIV) period included the above criteria plus those who received antenatal care at the facility, had a partner whether married or cohabiting, and were willing to pass on the partner invitation card as part of PA strategy to their partner.

### Sample size

The study had a sample size of 204 participants of which 110 were records reviewed in the pre-intervention period and 94 were participants enrolled in the MI in the EID period. The sample size was based on records that were found six months before and during the study intervention. We did not calculate the sample size because we were comparing what was happening six months before and after the implementation of MI in EID. The participants were enrolled consecutively if they met the eligibility criteria.

### Participant recruitment

Recruitment of participants in the intervention period started in March 2019 to August 2019 while implementing MI in EID. An HIV positive pregnant woman was informed of the study on admission to the delivery room (Labour ward). After delivery, her willingness to participate in the study was established; a research midwife in the Labour ward obtained informed consent, this midwife was not involved in the provision of care of the woman from Antenatal through labour and delivery period. Thereafter the woman was given a sealed envelope containing a partner invitation slip to give her partner. The participants were allowed to withdraw at any point of the study; all women who refused to participate in the study were assured that their normal medical care would not be affected in any way and women who did not participate in the study received the same care offered to participants.

### Follow up

Women who were given partner invitation cards as part of the PA strategy were only followed at 6 weeks from the enrollment.

### Data collection

We reviewed the maternity and EID of HIV registers at the facility to determine the EID of HIV uptake. Using a data extraction tool, data collectors captured the infant’s date of registration for the EID of HIV test and result. Therefore, to determine EID uptake on monthly basis we recorded infants who had an EID visit at six weeks of age who delivered at the facility as numerator against HIV positive women who delivered at the facility with a live neonate in the same period as denominator. We also captured the demographic characteristics of the HIV-positive women; age, education level, and parity.

The data extraction tools were validated by comparing the information on the tool to our objective to ensure that all parameters that were to be obtained to achieve the objective were there. The tool was piloted in extracting data that has not been used in this study and was amended to capture relevant information [32]. At the end of each month, the PI checked the data extraction tool for completeness and consistency and provided feedback to data collectors where necessary. The data were then entered in a password protected laptop using Microsoft excel 2010, it was a single-blinded data entry of the results to ensure validity and cater for mistakes. The data were then screened and cleaned to determine outliers, inconsistence, and strange patterns in the distribution to determine completeness. The excel sheet was then imported into STATA version 14 for analysis.

### Data analysis

We applied descriptive analysis to describe our data. Variables that were analyzed were; education level, age, parity, MI, and EID of HIV uptake. EID of HIV uptake was calculated based on the number of infants on whom EID of HIV was carried out at 6 weeks of age in a specific month against the HIV positive women who delivered at the facility in that month. Data analysis focused on describing the demographic characteristics of women in the non-intervention and intervention groups. The median and interquartile ranges (IQR) were used for continuous variables where appropriate while categorical variables were summarized using proportions. The variable of uptake EID of HIV services and Intervention (Male involvement) were tested using chi-square. We further calculated crude odds ratios because the variable of uptake EID of HIV was not dependent on the demographic characteristics of women.

## Results

**Chart 1:**
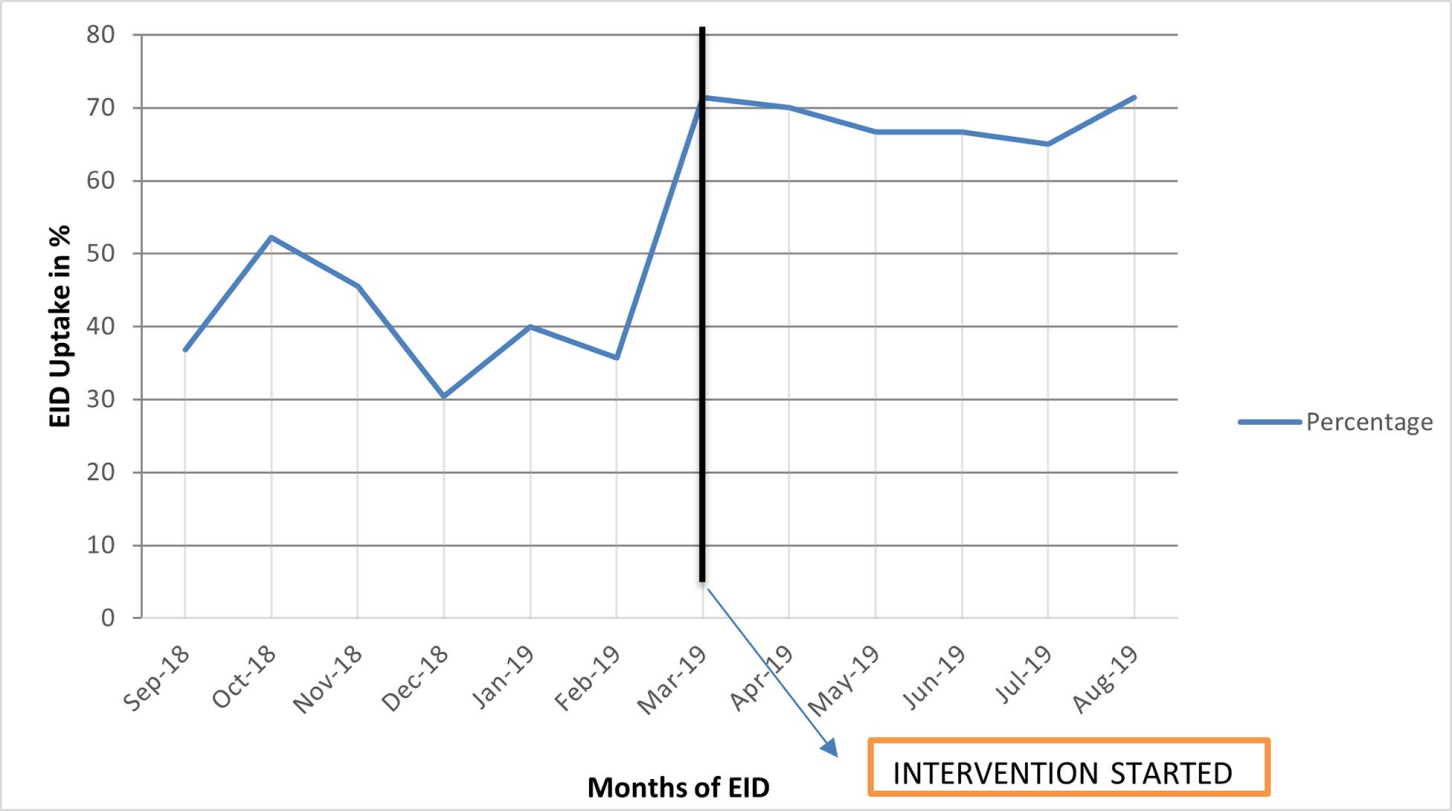
The proportion of uptake of EID on monthly basis for 12 months. Chart 1 above compares proportions of the uptake of EID in months before and during the implementation of MI in EID using PA strategy. The black line is the month when the intervention started.

**Fig 2:**
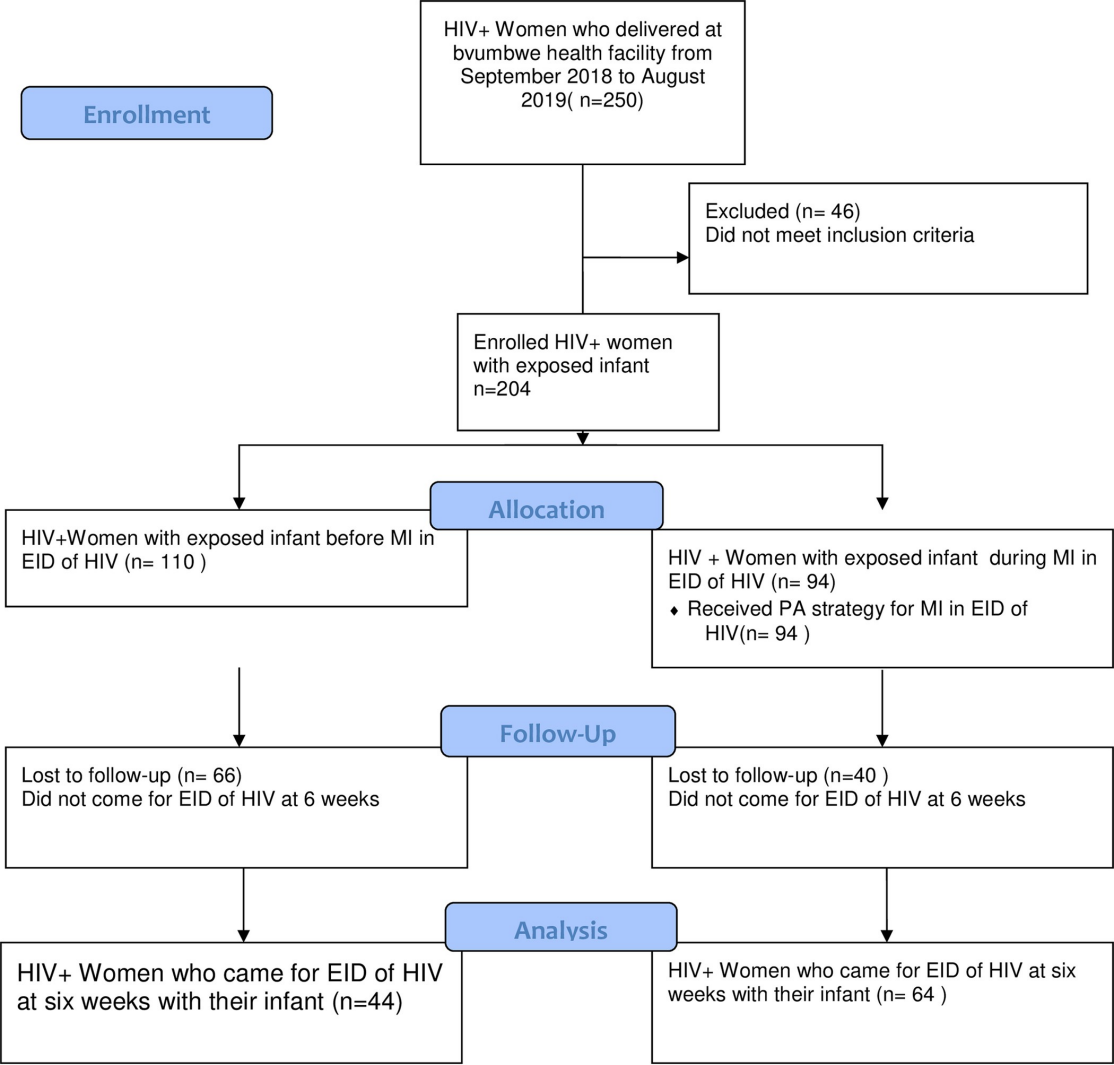
The flow of study participants in early infant diagnosis of HIV from September 2018 to August 2019 at Bvumbwe health facility.

There were 1,052 deliveries at Bvumbwe health facility from September 2018 to August 2019. Of these, 204(19.25%) were eligible for the study (Fig 2). The study recruited 94 participants who were eligible between March to August 2019 when MI in EID was being implemented and reviewed 110 records of HIV positive women who had delivered between the periods of September 2018 to February 2019 before MI in EID. Therefore, the total number of participants was 204 (Fig 2). Chart 1 below also shows a comparison of proportions of the uptake of EID in 6 months before and during the implementation of MI in EID using PA strategy.

### Social demographic characteristics of participants

Baseline socio-demographic, obstetrical characteristics were comparable between the two groups (Table 1). The median age of the women in the intervention group (MI) was 28 (IQR: 23–35) years and 26 (IQR: 22–33) years in the group before the intervention. The highest level of education was primary school education in both groups with 47 (50.00%) in the MI in the EID group and 53 (48.18%) in the period before MI in EID. In both the MI in EID and the period before MI in EID, most of the participants were multipara.

**Table 1:** Social demographic characteristics of women associated with male involvement in early infant diagnosis of HIV/AIDS.

	Before MI in EID	After MI in EID	P values
All clients n (%)	110 (53.92%)	94 (46.08%)	
**Age** Median (IQR)	26 (22–33)	28(23–35)	0.537
**Education status** None Primary Secondary	30(27.27%) 53(48.18%) 27(24.55%)	21(22.34%) 47(50.00%) 26(27.66%)	0.449
Parity Prim-Para (first delivery) Multipara (2–4 deliveries) Grand-multipara (5 and above deliveries)	23(20.91%) 72(65.45%) 15(13.64%)	13(13.38%) 66(70.21%) 15(15.96%)	0.135

### Uptake of early infant diagnosis of HIV services

The uptake of EID of HIV services in this study was defined as the proportion of HIV-exposed infants who received PCR test at six weeks post-delivery. Of the 204 participants enrolled in the study 108 (52.94%) took up the services. In the period before MI in EID, the uptake was 44/110 (40%) while in the MI in the EID period it was 64/94 (68.1%) (Fig 2).

### Social demographic characteristics of participants who came for EID of HIV at 6 weeks before and after the implementation of MI in EID of HIV and those who did not come

The socio-demographic and obstetrical characteristics of women who came for EID of HIV at 6 weeks and those who did not were comparable (Table 2). The median age of the women in the intervention group (MI) who came for EID of HIV at 6 weeks was 28 (24–34) years and 27(22.5–32) years in the group before the intervention. The highest level of education was primary school education in both groups who came for EID of HIV at 6 weeks with 22 (50.00%) in the MI in EID group and 28 (43.75%) in the period before MI in EID. In both the MI in EID and the period before MI in EID, most of the participants who came for EID of HIV at 6 weeks were multiparas.

**Table 2:** Social demographic characteristics of participants who came for EID of HIV at 6 weeks before and after the implementation of MI in EID of HIV and those who did not come.

EID UPTAKE Before MI = n(110) EID UPTAKE After MI n = (94)						
	Yes n = 44	No n = 66	Pvalue	Yes n = 64	No n = 30	pvalue
**Age**						
	27(22.5–32)	25(22–34)		28(24–34)	28.5(23–35.5)	
**Parity**						
1(one delivery)	7(15.91%)	16(24.24%)	0.539	8(12.50%)	5(16.67%)	0.840
2(2–4 deliveries)	30(68.18%)	42(63.64%)		46(71.88%)	20(66.67%)	
3(5 or more deliveries)	7(15.91%)	8(12.12%)		10(15.63%)	5(16.67%)	
**Education Level**						
None	12(24.07%)	18(27.27%)	0.928	14(21.88%)	7(23.33%)	0.088
Primary	22(50.00%)	31(46.97%)		28(43.75%)	19(63.33%)	
Secondary	10(22.73%)	17(25.76%)		22(34.38%)	4(13.33%)	

### Univariate analysis

Age, education level, and parity were not statistically significant with the uptake of EID of HIV among the women as seen in Table 3 as such we did not do multivariate analysis. However, the uptake EID of HIV remained higher with MI, odds Ratio of 3.2 (95%CI: 1.8–5.7) P = 0.001) compared to the uptake EID of HIV before MI OR of 0.6(95%CI: 0.46–0.98) P = 0.037

**Table 3:** Crude odds ratios for association between the social demographic characteristics of women and the uptake EID of HIV.

Variable	ODDS Ratio	95% Confidence Interval	P Value
**Age** 18–24 25–31 32–38 39–45 **Education level** None Primary Secondary **Parity**	0.7 1.13 0.7 0.9 1.37	0.33–1.47 0.56–2.28 0.19–2.51 0.44–1.8 0.61–3.08	0.351 0.720 0.592 0.751 0.441
Para 1(1 delivery) Para 2 or more (2 and above deliveries) **EID of HIV uptake** EID uptake before MI EID uptake in MI	1.57 0.6 3.2	0.73–3.34 0.4–0.98 1.8–5.70	0.244 0.037 0.001

## Discussion

Our study aimed at comparing the uptake of EID of HIV in the period before and after the implementation of MI using the PA strategy. We found out that the uptake of EID of HIV increased from 40% before implementation of Male involvement in EID to 68.1% during the implementation period and the association between the uptake of EID and male involvement was significant statistically OR 3.2 (95%CI: 1.7–5.5) P = 0.001). This meant that male involvement as an intervention in EID of HIV has the potential of increasing uptake of the service at 6 weeks by infants.

The findings of this study are similar to the findings of other studies that have determined that male involvement in services such as ANC and PMTCT of HIV tend to increase uptake and adherence to PMTCT intervention [8,15,16,33]. A study that was done in Kenya to establish an association between male antenatal attendance, HIV testing, and decreased Infant HIV infections showed that vertical transmission risk was lower among women with partner attendance compared to those without [34]. Among infants born to women with male attendance, the incidence of HIV infection was 16.30 per 100 person-years (95% CI 10.13–26.22) versus 30.86 per 100 person-years (95% CI 24.20–39.35) among those born to mothers without partner attendance [34]. This evidence provides a background for Malawi as a country to continue including men in EID of HIV services since MI has the potential of optimizing uptake of intervention that will reduce HIV infections among infants born to HIV positive mothers.

In contrast to the findings of our study, a study in Cambodia found that the uptake of PMTCT interventions was not influenced by male partner involvement but rather by maternal basic knowledge of HIV [35]. Mothers who had more knowledge considered the benefits of HIV testing which was followed with acceptance of the service unlike those without any knowledge [35]. In Cambodia, all women are involved in household decision-making [36] hence male partner involvement might not influence the uptake of service unlike in Malawi where men are the core decision-makers [37].

We also found out that maternal age, education level, and parity did not have any effects on the uptake of EID of HIV services at 6 weeks. This finding is consistent with a study in Cape town which found that maternal age and parity were not associated with the uptake of EID of HIV services at 6 weeks [38]. But they differ with a study in Kenya which found that mothers with less formal education were found to be at increased risk of reporting late for EID of HIV services unlike those who went up to secondary level [39]. Women with higher education are considered to comprehend well health educations on the EID of HIV. Other evidence suggests that delivery at a public facility, high maternal knowledge on PMTCT is a determinant to the uptake of EID of HIV [40] however, our study did not assess their effect on EID uptake before and after implementation of MI in EID of HIV due to the small sample size.

Our study has provided potentially relevant intervention and guidance towards the attainment of the first 90 target which states that 90% of the population should know their status by 2030. Our findings can help the Ministry of Health, HIV/AIDS Organisations, and programme implementers to direct appropriate intervention to help in increasing timely uptake of EID of HIV at six weeks. Further studies should continue to investigate the impact of MI in EID of HIV uptake using robust designs to add to the strength of evidence already available.

### Study strengths and limitations

Our study has provided a potential intervention, MI that could increase the uptake of EID of HIV at 6 weeks. It has also provided strategies that can be used to improve male participation in EID and PMTCT services. The study also faced some limitations as follows; we cannot draw a causal inference because we used a quasi-experimental before and after design and we did not calculate the sample size. The period of the study may also constitute a limitation because though we compared six months before to six months after, the time trends were different hence prone to chronological bias.

### Conclusion

Uptake of EID of HIV at six weeks increased compared to the period before MI in EID. This showed that male involvement in the EID of HIV services is a potential intervention that could increase EID uptake in our setting. Also, maternal age, education levels, and parity did not have any impact on the uptake of EID of HIV services. Therefore, we recommend a more robust randomised control trial on the uptake of EID of HIV services to add in to the strength of evidence available, in optimizing uptake and reach the goals set by the Malawian PMTCT/ART guidelines.

## Supporting information

S1 Appendix Partner invitation card. (PDF)
