# Supplementary material for: Male involvement enhances the uptake of early infant diagnosis of HIV services in Thyolo, Malawi: A non-equivalent control group quasi-experimental study
Source: PLoS One. 2023 Feb 22;18(2):e0281105. doi: 10.1371/journal.pone.0281105 (PMC9946214; doi:10.1371/journal.pone.0281105)
Supplement: S1 Appendix — (PDF) [file pone.0281105.s001.pdf]

INVITATIONS FOR MALE PARTNER

INVITATION CARD

TO……………………………………………………………………………………….

YOUR WIFE/PARTNER IS ACCESSING POSTNATAL CARE AT BVUMBWE HEALTH CENTER; WE ARE INVITING YOU TO ACCOMPANY HER AT SIX WEEKS POSTNATAL VISIT SO THAT WE COUNSEL BOTH OF YOU ON THE HEALTH OF THE BABY.

IF YOU HAVE ANY QUESTIONS CALL MISS MIRIAM WINDOW ON THIS NUMBER 0884523963 OR ASK ANY NURSE AT BVUMBWE HC

Chichewa version

KALATA YOKUITANANI KUCHIPATALA

Ikupita kwa…………………………………………………..

Akazi anu akulandila chithandizo pamodzi ndi mwana wanu atabeleka kuno kuchipatala chanching'ono cha Bvumbwe. Choncho tikukupemphani kuti muzabwele nao limodzi pamene akuzalandilanso chithandizochi pamodzi ndi mwana wanu pakatha sabata zisanu ndi imodz kuti muzalandile uphungu okhunza ndi umoyo wa mwana wanuyi.

Ngati mulindi mafunso ena alionse mutha kuimbira a Miriam window panambala iyi 0884523963 kapena kufunsa kwa aliyense ogwila ntchito kuchipatala chaching'ono cha Bvumbwe.
